# Role of HLA-G in Viral Infections

**DOI:** 10.3389/fimmu.2022.826074

**Published:** 2022-02-14

**Authors:** Simon Jasinski-Bergner, Dominik Schmiedel, Ofer Mandelboim, Barbara Seliger

**Affiliations:** ^1^ Institute of Medical Immunology, Martin Luther University Halle-Wittenberg, Halle (Saale), Germany; ^2^ Department of Good Manufacturing Practice (GMP) Development & Advanced Therapy Medicinal Products (ATMP) Design, Fraunhofer Institute for Cell Therapy and Immunology (IZI), Leipzig, Germany; ^3^ The Lautenberg Center for General and Tumor Immunology, The BioMedical Research Institute Israel Canada of the Faculty of Medicine, The Hebrew University Hadassah Medical School, Jerusalem, Israel

**Keywords:** human leukocyte antigen G, viral infection, immune escape, virus-induced tumors, interleukin 10

## Abstract

The human leukocyte antigen (HLA)-G is a non-classical HLA class I molecule, which has distinct features to classical HLA-A, -B, -C antigens, such as a low polymorphism, different splice variants, highly restricted, tightly regulated expression and immune modulatory properties. HLA-G expression in tumor cells and virus-infected cells, as well as the release of soluble HLA-G leads to escape from host immune surveillance. Increased knowledge of the link between HLA-G expression, viral infection and disease progression is urgently required, which highlights the possible use of HLA-G as novel diagnostic and prognostic biomarker for viral infections, but also as therapeutic target. Therefore, this review aims to summarize the expression, regulation, function and impact of HLA-G in the context of different viral infections including virus-associated cancers. The characterization of HLA-G-driven immune escape mechanisms involved in the interactions between host cells and viruses might result in the design of novel immunotherapeutic strategies targeting HLA-G and/or its interaction with its receptors on immune effector cells.

## Introduction

Accumulating evidence exists that immune suppressive mechanisms play a critical role in promoting viral infections by either suppressing the capacity of infected host cells to overcome viral infection or by preventing the elimination of virus-transformed cells by immune effector cells. A common mechanism to escape immune surveillance is a loss or downregulation of classical HLA class Ia antigens and the neoexpression of non-classical HLA class Ib antigens, such as HLA-E, -F and –G (1–3). While the expression of HLA class Ia antigens leads to a T cell-mediated control of host immune responses mediated by antigen presentation and recognition, neoexpression of HLA-G has immune modulatory properties by inhibiting both innate and adaptive immune responses thereby leading to an immune escape of virus-infected cells. Although diverse viruses exploit HLA-G to establish persistent infections, the underlying molecular mechanisms tremendously differ. In this review, the expression, regulation, and clinical impact of HLA-G neoexpression in the context of viral infections as well as the underlying molecular mechanisms will be summarized.

## Features of the HLA-G

The non-classical HLA-G gene is located on the short arm of chromosome 6 and consists of 8 exons, 7 introns, a 5’ upstream regulatory region (URR) and a 3’ untranslated region (UTR). The primary HLA-G transcript is alternatively spliced into at least 7 mRNAs, which encode 4 membrane-bound (HLA-G1, -G2, -G3, -G4) and 3 soluble (sHLA-G; HLA-G5, -G6, -G7) protein isoforms (4). Each HLA-G isoform contains 1-3 extracellular domains (α1, α2, α3), which are encoded by exon 2, 3 and 4; exons 5 and 6 encode the transmembrane and cytoplasmic domain of the heavy chain (HC), while the presence of intronic sequences are highly variable (5). The HLA-G1 and sHLA-G5 isoforms have a comparable structure to classical HLA class Ia antigens (HLA-A, -B, -C) and contain the HLA class I HC, which is non-covalently linked to β_2_-microglobulin (β_2_-m) (6–9). Furthermore, a peptide is bound in the cleft of the α1 and α2 domains, while the α3 domain of both the membranous and the sHLA-G is bound by the CD8 co-receptor (10). Similar to the classical HLA class Ia molecules, HLA-G is capable of presenting a broad repertoire of peptides and requires peptide binding to be efficiently presented on the cell surface (11–13). The other HLA-G isoforms lack one or two extracellular domains, are smaller and not associated with β_2_-m. HLA-G1 to HLA-G4 are membrane-bound and have a transmembrane region. In contrast, HLA-G5 and HLA-G6 are soluble isoforms with the presence of intron 4, which contains a premature stop codon thereby preventing the translation of the transmembrane and cytoplasmic residue. HLA-G7 is also a soluble isoform due to the presence of intron 2 containing a pre-mature stop codon. Recently, a number of novel HLA-G isoforms have been identified, which could be generated by skipping exon 6 and by a deletion of the α1 domain (8, 14). It should be noted that sHLA-G1 could be generated by a metalloproteinase-dependent shedding (15). Furthermore, membrane-bound HLA-G can also circulate within the blood stream, in particular in form of extracellular vesicles (10).

## Physiological Expression and Immune Suppressive Activity of HLA-G

**Figure 1:**
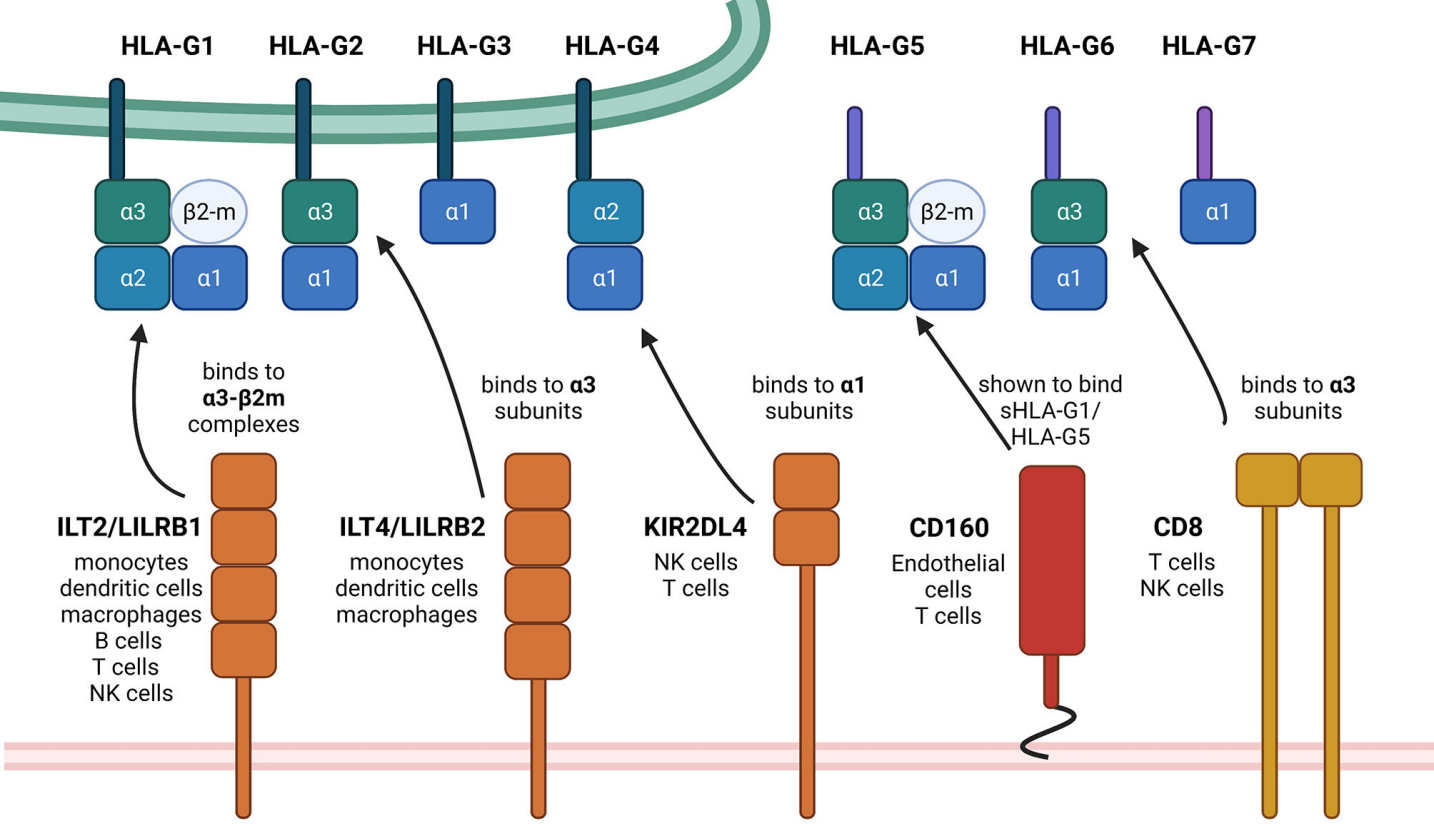
Summary of HLA-G isoforms with their binding positions for the so far known receptors. This schematic summary of the structural differences of HLA-G isoforms shows the so far known HLA-G receptors and also designates the exact binding position. The black arrows show exemplarily, but not exclusively possible interactions, depending on mentioned binding positions on the HLA-G ligands. There exist first evidence of a possible interaction between NKG2A with HLA-G, which still needs to be investigated in more detail. Therefore, NKG2A is not yet mentioned within this scheme. Created with BioRender.com.

HLA-G expression is generally restricted to immune privileged tissues, such as cytotrophoblasts, cornea and pancreatic islets (16–18). However, HLA-G neoexpression was detected under pathophysiological conditions, such as cancers, inflammatory diseases, auto-immune diseases and pathogen infections including viruses (19). It is known that HLA-G has immune suppressive properties by interacting with different inhibitory receptors, namely ILT2/LILRB1, ILT4/LILRB2 and KIR2DL4, which are expressed on various immune cell subpopulations (20–22). Other receptors have been recently discovered, which are able to bind HLA-G, in particular NKG2A (23), CD160 (24) and CD8 (25) thereby interacting with the α3 domain of the respective HLA-G isoforms. The structures of the HLA-G isoforms, their receptors including their HLA-G-binding domains and the cell types expressing these receptors are summarized in **Figure 1**. The interaction of these receptors with HLA-G leads to the inhibition of the cytolytic function of natural killer (NK) cells and CD8^+^ cytotoxic T lymphocytes (CTLs), macrophage-mediated cytotoxicity, allo-proliferative response of CD4^+^ T cells and of the maturation as well as function of dendritic cells (DCs).

However, it needs to be underlined that the interactions of HLA-G with immune effector cells are even much more diverse and complex as summarized so far, since also indirect immune modulatory effects of HLA-G on immune effector cells are known. Recently, it could be demonstrated that the non-classical HLA-E molecule can present peptides derived from HLA class Ia, but also from HLA-G leader sequences (26–30). HLA-E is a ligand for the inhibitory CD94/NKG2A,-B receptors and for the activating CD94/NKG2C receptor expressed on NK cells (31). This indirect mechanism modulates the immune effector mechanisms depending on the context of the engaged receptors. For example, the HLA-G-derived nonamer VMAPRTLFL presented on HLA-E molecules caused an enhanced NK cell-mediated lysis in an *in vitro* experiment of transfected 721.221 cells naturally lacking HLA class Ia/b molecules thereby representing valuable targets for NK cells. However, it needs to be addressed in much more detail whether this effect also plays a role *in vivo*, especially in the context of sHLA-G molecules within the TME of solid tumors inhibiting immune effector cells even prior to tumor infiltration.

In addition, immune suppressive cells are stimulated to secrete cytokines, like transforming growth factor (TGF)-β and interleukin (IL-10), which are able to increase HLA-G expression (32–35). Furthermore, the interaction of HLA-G with its receptors could lead to long-term immune modulatory effects by inducing and/or accumulating regulatory T cells (Tregs) (36), mesenchymal stem cells (MSCs) (37, 38) and myeloid-derived suppressor cells (MDSCs) (39, 40). In addition to the direct interaction of HLA-G with its appropriate receptors, the HLA-G-mediated immune suppression could be also caused by intercellular transfer mechanisms, such as trogocytosis, exosomes or tunneling nanotubes, which also leads to escape from the destruction by the host immune system.

## Polymorphisms Affect the Expression of HLA-G

So far, more than 88 different HLA-G alleles (41) have been discovered. Within its coding region, HLA-G shows a limited protein variability compared to classical HLA class Ia, but both the 5’ URR and the 3’ UTR contain a multitude of polymorphic sites affecting gene regulation (42). The major HLA-G polymorphisms associated with viral infections and their role in virus susceptibility are summarized in **Table 1**. Response elements for diverse transcriptional inducers have been identified, like the progesterone response elements (PRE), heat shock elements (HSE), interferon-stimulated response elements (ISRE) and elements responding to cyclic AMP (CRE) (50–54). In addition, negative regulators have been identified like the ras-responsive elements (RRE) (55). Many polymorphisms and single nucleotide variants within the regions of such response elements have been described (56, 57). *In vitro* evaluations of the effects of the most frequent 5’ URR haplotypes have indeed shown differential transcriptional regulation of HLA-G (58, 59). In addition, to the 5’ UTR, diverse polymorphic sites exist also in the 3’ UTR of the HLA-G transcript (60); many of them have been shown to change the affinity of gene-targeted sequencing for transcriptional or post-transcriptional factors, affecting splicing (61), microRNA (miRNA) binding (62) and RNA turnover (63). A variation of major significance for HLA-G expression is a 14 bp insertion/deletion (ins/del, rs66554220) in the 3’ UTR, which is associated with the alternative splicing of HLA-G and miRNA stability. Furthermore, the region downstream of the 14 bp ins/del polymorphic site has been suggested to be a target for HLA-G-specific miRNAs thereby leading to a direct downregulation of HLA-G. However, there exist controversial data regarding the role of the HLA-G 14 bp ins/del polymorphism and susceptibility to viral infections (64). Of note, population studies suggest that HLA-G haplotypes are strongly shaped by selective pressure throughout evolution thereby preserving protein-coding sequences and enabling divergence in regulatory sequences (42, 65).

**Table 1:** Impact of HLA-G polymorphisms on viral infections and virus-mediated diseases.

Virus	Polymorphism/allele	Regulatory impact
HPV	+14 bp/+14 bp	significant association with an increased risk for HPV18 infection (OR = 12.95, P < 0.01) (43)
	+14 bp/-14 bp	increased risk for HPV58 infection (OR = 5.55, P < 0.05) (43)
	+14 bp/G	significant increase in HPV18-infected patients (60.0% vs 27.3%, OR = 4.00, Pc < 0.05) (43)
	frequency of the allele +3142 C	significant decrease in HPV-infected patients compared with normal controls (43)
	frequency of the genotype +3142 CC	significant decrease in HPV-infected patients compared with normal controls (43)
	130C del	association with an increased progression and reduced overall survival of NPC patients (44)
CMV	+3142 CC genotype	association with a higher susceptibility to CMV infection after kidney transplantation (45)
HIV	HLA-G*010108 allele	association with a 2.5-fold increased risk of HIV-1 infection (46)
	14 bp del/del	significantly reduced rates of perinatal HIV transmission (47)
HTLV-1	14 bp del/del	higher proviral load (48)
SARS-CoV-2	SNP rs9381042	correlation with severe COVID-19 infections (49)

## Regulation of HLA-G Expression and Viral Infections

There exist more than 220 viral species able to infect humans (66) and annually even more human pathogenic viruses are identified or even emerge including the severe acute respiratory syndrome coronavirus 2 (SARS-CoV-2) (67). In 2008, before the SARS-CoV-2 pandemic started, Woolhouse and co-authors generated *in silico* prediction tools for estimating the emergence of novel human pathogenic viruses concluding that the formation/discovery of novel human viruses must be anticipated in public health planning and the future should prove this statement (68).

Infection with intracellular pathogens, such as viruses, could lead to the presentation of pathogen-relevant peptides, e.g. *via* HLA class I molecules to T cells resulting in T cell activation and elimination of the infected host cell, although numerous other molecular mechanisms also contribute to anti-viral immune surveillance. These include the induction of the MICA/MICB molecules as well as the UL16-binding proteins (ULBP)1-6, which are physiologically not expressed, but induced by cellular stress and viral infections and act as ligands for the activating NKG2D receptor present on NK cells and CTLs (69). In addition, intracellular, membranous and even secreted pattern recognition receptors exert a strong role in anti-viral immune surveillance. A prerequisite for appropriate immune effector cell functions is the recruitment of these immune effector cells to the virus-infected cells, a process, which involves a functional chemokine signaling. Indeed, the authors recently reviewed the distinct viral mechanisms targeting the above mentioned strategies involved in immune recognition and elimination of viral infections (70).

However, several human pathogenic viruses can successfully develop immune evasion by various molecular mechanisms, including the induced expression of immune checkpoints, like PD-L1 and HLA-G, in the virus-infected host cells (1, 71–73). In this review, we will summarize known examples of human viruses able to induce immunological tolerance towards host immune effector cells by HLA-G induction. Therefore, an overview of the tightly regulated HLA-G expression will be provided.

HLA-G is predominantly expressed in immune-privileged tissues, in particular placental and eye tissues (17, 74), but also insulin- and glucagon-positive cells within the endocrine islets of the pancreas (18) and the medullary thymic epithelial cells (75) and locally induce immunological tolerance thereby preventing tissue damages due to inflammatory reactions. The HLA-G gene expression itself is regulated at multiple levels, including the grade of promoter methylation (76), the acetylation of histones (77), the existence of transcriptional activators (CREB1, AIRE) (78, 79) as well as the lack of transcriptional repressors (RREB-1, LINE1) (55, 80). In addition to the transcriptional control, several factors contribute to a complex posttranscriptional gene regulation of HLA-G including many miRNAs, such as miR-148 family members with the highest affinity to the 3’ UTR of the HLA-G transcript and miRNA directed against the coding sequence (CDS) of HLA-G (81, 82) as well as long non-coding RNAs like HOTAIR (83). Furthermore, RNA-binding proteins (RBPs), like e.g. HNRNPR, have been reported with regulatory potential for the HLA-G mRNA (84).

Several cytokines, but also certain stress stimuli are known to enhance the HLA-G levels, like interferon (IFN)-γ, IL-10, TGF-β (76), hypoxia and heat stress (52, 85).

This complex regulatory network for the control of the HLA-G expression can be altered to induce or enhance the immune evasion of tumor cells, but also of infectious pathogens, e.g. viruses, bacteria and parasites (86). It is noteworthy that a number of viruses have an oncogenic potential and independently of viral infections a pathophysiological HLA-G neoexpression, which can be detected with high frequencies in virus-associated malignancies as well as independently of viral infections in solid and hematopoietic tumor diseases (87–90).

## Role of HLA-G in Viral Infections – Expression, Mechanisms and Clinical Relevance

### HLA-G and Human Papillomavirus

Human papillomavirus (HPV) infection is a common infection and has been linked to epithelial cancers including in particular cervical and head and neck cancers (91–93). HLA-G has an impact on the clinical course of persistent HPV infections, epithelial cell transformation, tumor growth, metastasis, formation and therapy resistance. In HPV-associated tumors like cervical cancer and head and neck squamous cell carcinoma (HNSCC), a role of HLA-G in HPV infections and in the initiation and progression has been described (94, 95) mediated by polymorphisms, methylation and deregulation of HLA-G (44).

HLA-G polymorphisms are genetic susceptibility and/or disease-relevant factors for cervical HPV infections and viral persistence of cervical cancers (**Table 1**). Most of the studies focused on the polymorphisms in the 3’ UTR of the HLA-G gene rather than on its promoter region or in its CDS. The HLA-G +14/G and +3142G alleles are risk factors for HPV infections and increase the risk of high-grade cervical lesions and are associated with cervical carcinogenesis (43). Interestingly, the HLA-G promoter methylation was not associated with the HPV infection status (96). Moreover, a codon 130C deletion was associated with an increased progression and reduced overall survival of patients with nasopharyngeal carcinoma (NPC) (97).

In comparison to HPV-negative HNSCC, higher HLA-G expression levels were found in HPV-positive HNSCC with HLA-G7 as the most frequent isoform (98). In addition to various HLA-G polymorphisms, which have been associated with the susceptibility of HPV infection, an influence of HLA-G on the immune modulation of HPV-positive HNSCC has been described. Furthermore, high levels of IFN-γ, but lower levels of IL-10, TGF-β, SOCS1/3 and programmed death receptor 1 (PD1) were found in HPV-positive HNSCC (98).

### HLA-G and Hepatitis Virus

Infections with hepatitis B (HBV) and C virus (HCV) are major health threats worldwide (99, 100). Chronic HBV/HCV infection is followed by chronic hepatitis, which might lead to liver cirrhosis and hepatocellular carcinoma (HCC) (101). Despite the host immune response is crucial for the control of HBV and HCV, both viruses have developed different strategies to escape immune surveillance including HLA-G neoexpression (102). Indeed, a high frequency of HLA-G expression was found in tissues of HCV-infected patients. In addition, elevated sHLA-G serum levels were detected in patients with chronic HCV infection (103, 104). Furthermore, HLA-G expression was more prominent in high fibrosis specimens. In association with the increased HLA-G expression in liver, an enhanced inflammatory activity and liver fibrosis were demonstrated, which might have implications for progression and prognosis of liver diseases. HLA-G expression in the liver upon chronic HCV infection might be due to the composition of the inflammatory infiltrate, in particular of mast cells known to induce fibrosis by stimulating the proliferation of TGF-β-secreting hepatic stellate cells as demonstrated by Amiot and co-authors (105). In addition, hepatic stellate cells also secrete IL-10, which is able to upregulate HLA-G expression. Thus, the liver microenvironment including cytokines affecting the inflammatory response and fibrosis, such as IL-10 and TGF-β, are responsible for the induction of liver HLA-G expression in chronic hepatitis-diseased patients (104).

As described for HCV-infected tissues, HLA-G is expressed at a high frequency in HBV-infected liver biopsies, but not in control hepatocytes (106). Furthermore, sHLA-G levels were also significantly higher in HBV-infected patients when compared to healthy controls (15, 107, 108). The HBV-mediated induction of HLA-G expression in hepatocytes might be caused by modulation of the hepatocyte expression of miRs, such as downregulation of the HLA-G-regulating miR-152 representing the most HLA-G mRNA affine member of the miR-148 family leading to neoexpression of HLA-G (109). These data further suggest that HLA-G and HLA-G-specific miRs are involved in HBV-induced HCC.

### HLA-G and Human Cytomegalovirus

Human cytomegalovirus (HCMV) causes a life-long human infection, which may be life-threatening for immune-suppressed patients (110, 111). Like other viruses, HCMV has developed different strategies to escape immune surveillance including the modulation of HLA-G expression (112). Membrane-bound HLA-G was shown to be expressed in macrophages and monocytes undergoing lytic infection, while sHLA-G levels are increased in serum of patients with acute HCMV infection (113–115). Furthermore, an enhanced HLA-G expression has been associated with allograft tolerance after kidney transplantation (116). The single nucleotide polymorphism (SNP) (3142C>G) in the HLA-G gene of the recipient, but not in the transplant donor was associated with a higher susceptibility to HCMV infection after kidney transplantation. In addition, sHLA-G levels were associated with a higher susceptibility to HCMV infection (**Table 1**). These data suggested that both the recipient HLA-G+3142CC phenotype and sHLA-G levels represent predictive risk markers for HCMV infection (45). Recently, an association between other HLA-G 3’ UTR variants and kidney graft outcomes has been reported. In recipients with stable allograft function, significantly higher sHLA-G levels were found in patients who were +3010GG, +3142CC, +3187GG and +3196CC carriers in comparison to acute rejection recipients (117). Thus, there exists a direct association between this HLA-G 3’ UTR variants and sHLA-G levels in kidney recipients leading to graft acceptance. Therefore, monitoring of sHLA-G levels prior to transplantation might serve as suitable marker to predict kidney graft outcome. This was confirmed by a recent study demonstrating that the acute and chronic rejection rate of kidneys increased 1.06 times and 1.14 times, respectively, in kidney transplant recipients with low serum sHLA-G levels. The frequency of acute rejection was lower in patients with a 14 bp del/del polymorphism than that of ins/ins and ins/del polymorphisms. Based on these results, the HLA-G 3’ UTR polymorphism and the sHLA-G levels might represent useful markers for the prediction of rejection in kidney transplants (118).

### HLA-G and Human Herpesvirus 6

Human herpesvirus (HHV)-6 is a β-herpesvirus comprising of the two viruses HHV-6A and HHV-6B (119–121) that cause both productive and life-long latent infections (122). HHV-6A/B induce HLA-G expression in mesothelial cells leading to impaired NK cell functions against virus-infected cells (123). HHV-6A/B express the viral protein U94, which has key functions in the viral life cycle and elicits immune responses. Furthermore, U94 has been shown to induce HLA-G expression by upregulating the expression of the transcription factor ATF3, which activates HLA-G expression and release (124). In line with these findings, patients suffering from systemic sclerosis showed elevated sHLA-G levels when being co-infected with HHV-6A. Furthermore, viral load correlated to NK cell dysfunction and disease severity (125).

### HLA-G and Epstein-Barr Virus

Another member of the HHVs, the Epstein-Barr virus (EBV), has also been reported to induce the HLA-G expression by yet undefined molecular mechanisms (126). It could be speculated that the EBV-encoded viral IL-10 (vIL10), a known agonist of the human IL-10, might be an inducer of the HLA-G expression. Interestingly, EBV infections also affect the epigenetic control within the host cell genome, including altered DNA methylation patterns (127) as well as aberrant histone modifications (128), both mechanisms known as important regulators of HLA-G gene expression. It is noteworthy that EBV infections can be linked to various tumor entities, such as NPC, gastric adenocarcinoma (GC), classical Hodgkin lymphoma (cHL) and Burkitt lymphoma (BL) (129), which have been shown to exhibit pathophysiological HLA-G neoexpression.

### HLA-G and Human Immunodeficiency Virus

The human immunodeficiency virus (HIV) is a RNA virus. Several studies suggested elevated sHLA-G in serum samples of untreated HIV patients, but membrane-bound HLA-G is also detected on immune cells of patients. Furthermore, HIV infection is not only inducing HLA-G on monocytes, macrophages and T lymphocytes (130), but also causes an increased HLA-E expression in infected CD4^+^ T helper cells (131), which exerts immune modulatory functions comparable to that of HLA-G thereby contributing to immune evasion of HIV-infected cells (131). HLA-E is the ligand for the inhibitory NK cell receptors CD94/NKG2A, -B and for the activating -C expressed on NK cells and CTL (132). HIV infection also induces the expression of the immune modulatory ligands PD-L1 and PD-L2 on macrophages (133), which interact with their receptor PD-1. The PD-L1/PD-1 interaction causes an inhibition of CTL activity, but also an increased IL-10 secretion, which contributes to immune evasion of HIV-infected CD4^+^ T helper cells (134).

The HIV-related induction of HLA-G expression has been currently well characterized. Certain HLA-G alleles increase the risk for heterosexual transmission of HIV in African women. Indeed, the HLA-G*010108 allele was associated with a 2.5-fold increased risk of HIV-1 infection and the two HLA-G*010108 alleles containing genotypes HLA-G*010108/010401 and G*010101/010108 were associated with an elevated risk of HIV-1 infection (**Table 1**) (46). Furthermore, children carrying the homozygous HLA-G genotype with the 14 bp deletion within the HLA-G 3’-UTR exhibited significantly reduced rates of perinatal HIV transmission (47, 135).

The overexpression of HLA-G in HIV-infected individuals may be secondary due to an increased release of IL-10. The HIV-encoded protein gp41 has been identified to induce IL-10 secretion in monocytes (136) thereby revealing a possible mechanism for the occurrence of HLA-G.

In addition, HIV infection causes a downregulation of the classical HLA class Ia molecules HLA-A and HLA-B (137), a known mechanism for (tumor) immune evasion, clearly demonstrating a deregulation of appropriate HLA class Ia/b signaling upon HIV infection.

### HLA-G and Human T Cell Lymphotropic Virus Type I

The human T cell lymphotropic virus type 1 (HTLV-1) known to induce very aggressive adult T-cell lymphoma (ATL) also upregulates HLA-G expression, mainly HLA-G1 and HLA-G5 (138), and in analogy to HIV, the homozygous HLA-G genotype −14-bp/−14-bp genotype has a higher proviral load than the +14-bp/−14-bp and +14-bp/+14-bp genotypes (**Table 1**) (48). However, a detailed mechanism for the HTLV-1-mediated HLA-G increase has not yet been identified, but an IL-10-based mechanism analogous to the HIV-dependent HLA-G induction might be likely.

### HLA-G and Influenza A Virus

Influenza A virus (IAV) causes acute respiratory infections (139). Infections with IAV are known to induce HLA-G mRNA and protein expression in alveolar epithelial cells (140), which is a major inhibitory molecule of host immune responses to IAV infections. Although the detailed underlying mechanisms have not yet been determined, the study suggested an involvement of cytokines, in particular IFNs, which could cause the HLA-G neoexpression mediated by the viral encoded NS1 protein.

### SARS-CoV-2 and HLA-G

In 2019, a novel corona virus emerged that caused a worldwide pandemic and lead to the potentially life-threatening severe acute respiratory syndrome (SARS-CoV-2) (67). In patients with severe infection, high serum levels of sHLA-G were observed (141) and HLA-G was also found on the cell surface of diverse immune cells during the course of infection. HLA-G was detected on monocytes, T and B cells and showed a high-low-high pattern, probably correlating with infection, replication and clearance phase (142). However, these results were only compiled from a single patient and require further validation. Another clinical study found a correlation between high sHLA-G levels and improved disease outcome probably due to immune-dampening effects of HLA-G that suppress an excessive tissue-damage, possibly mediated by reduced neutrophil infiltration to the sites of infection (143). Disease outcome was not clearly linked to HLA-G variants despite a meta-analysis listed the HLA-G 3’UTR SNP rs9381042 as a candidate variant that was slightly overrepresented in a UK cohort of critically ill patients due to COVID-19 infection compared to the general population (**Table 1**) (49).

## Conclusions

**Figure 2:**
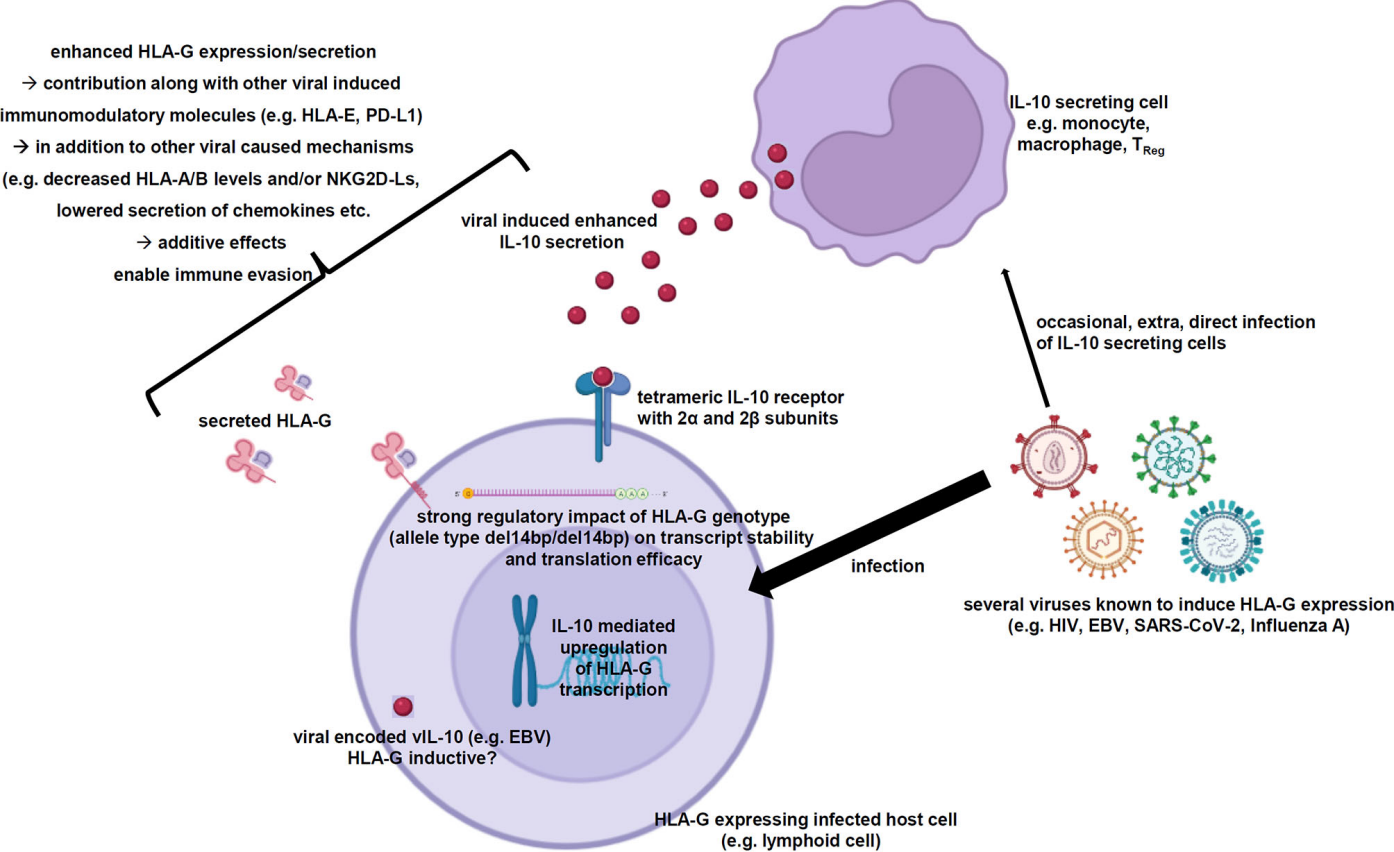
Scheme summarizing the virus-induced enhanced IL-10 secretion resulting in elevated HLA-G expression and secretion. The working hypothesis demonstrates the elevated IL-10 secretion of certain immune effector cells as consequence of viral infections, which is very likely the main driver of the increased HLA-G gene expression and secretion within the virally infected host cells strongly contributing to immune evasion. Created with BioRender.com.

As summarized in this review, multiple viral infections are known to induce membranous HLA-G expression as well as its secretion in infected host cells. In some cases, the role of HLA-G in infections has been characterized to contribute to immune evasion of the infected host cells by inhibition of immune effector cells. So far, the detailed molecular mechanisms of this HLA-G neoexpression have not yet been identified. In many of these studies a correlation of the HLA-G neoexpression with elevated IL-10 levels is reported. Indeed, IL-10 is a known inducer of the HLA-G expression and has also been described in tumor cells independently of viral infections as inducer of HLA-G expression. Therefore, the very likely IL-10-based hypothesis of the virus-driven HLA-G induction is summarized in **Figure 2**.

Physiologically, IL-10 exerts its functions as an anti-inflammatory cytokine in preventing inflammatory and autoimmune pathologies by inhibition of certain immune effector cells thereby preventing inflammation-induced and autoimmune pathologies (144). Murine *in vivo* models inhibiting the IL-10R demonstrated that the viral infection with lymphocytic choriomeningitis virus resulted in a rapid resolution of the persistent infection. This blocked IL-10 signaling was also linked to an increased IFN-γ secretion by CD8^+^ T cells (145). Another murine study with IL-10-deficient mice could also demonstrate a better anti-viral T cell response against the lymphocytic choriomeningitis virus (146).

The predominant IL-10-expressing cells are Th2 cells and Tregs, but also macrophages, dendritic cells, eosinophils and neutrophils are known to secrete IL-10 (144). Some of these immune cell populations were directly infected by HLA-G-inducing viruses and subsequently shifted towards increased IL-10 secretion. In other cases, indirect effects, like e.g. an increased PD-L1/PD-1 signaling, caused an enhanced IL-10 secretion of these immune effector cells.

Once the HLA-G gene transcription has been induced upon IL-10 stimulation, the homozygous genotype del14bp/del14bp has additional beneficial effects due to an enhanced HLA-G mRNA stability by avoiding miRNA-based downregulation and thereby resulting in increased HLA-G protein levels.

It is also known that IL-10 stimulation affects the RNA expression profile in IL-10-stimulated cells (147), but whether HLA-G-regulating miRs are downregulated upon IL-10 signaling is so far unknown. In addition, further direct or indirect effects of IL-10 on other HLA-G-regulating factors are rather limited with the exception of the transcriptional activator CREB1, which induces the transcription of HLA-G and IL-10 (78, 148). Thus, the regulation of HLA-G expression upon viral infection is complex and further studies are urgently needed to gain deeper insights into the molecular mechanisms of the viral infection-driven HLA-G neoexpression.

## Author Contributions

SJ-B and BS designed the study. SJ-B, DS, OM, and BS wrote the manuscript. SJ-B and DS created the figures. All authors contributed to the article and approved the submitted version.

## Funding

This work was funded by the German-Israeli Foundation (GIF; I-37-414.11-2016) and Dr. Werner Jackstädt Foundation.

## Conflict of Interest

The authors declare that the research was conducted in the absence of any commercial or financial relationships that could be construed as a potential conflict of interest.

## Publisher’s Note

All claims expressed in this article are solely those of the authors and do not necessarily represent those of their affiliated organizations, or those of the publisher, the editors and the reviewers. Any product that may be evaluated in this article, or claim that may be made by its manufacturer, is not guaranteed or endorsed by the publisher.

## Abbreviations

APC, antigen presenting cells; β_2_-m, β_2_-microglobulin; CDS, coding region, coding sequence; CTL, cytotoxic T lymphocyte; DC, dendritic cell; HC, heavy chain; HCMV, human cytomegalo virus; HCC, hepatocellular carcinoma; HCV, hepatitis C virus; HHV, human herpesvirus; HLA, human leukocyte antigen; HNSCC, head and neck squamous cell carcinoma; HPV, human papilloma virus; HTLV-1, human I-lymphotropic virus type II; IAV, influenza A virus; IL, interleukin; MDSC, myeloid-derived suppressor cell; miRNA, microRNA; MSC, mesenchymal stem cells; NK, natural killer; NPC, nasopharyngeal carcinoma; PD1, programmed death receptor 1; SARS-CoV-2, severe acute respiratory syndrome coronavirus 2; SNP, single nucleotide polymorphism; TGF-β, transforming growth factor β; Treg, regulatory T cell; ULBP, UL16-binding protein; URR, upstream regulatory region; UTR, untranslated region.
